# Control and prediction of the organic solid state: a challenge to theory and experiment^†^

**DOI:** 10.1098/rspa.2018.0351

**Published:** 2018-09-19

**Authors:** Sarah L. Price

**Affiliations:** Department of Chemistry, University College London, 20 Gordon St, London WC1H 0AJ, UK

**Keywords:** organic crystal structures, polymorphism, theoretical prediction, pharmaceutical development

## Abstract

The ability of theoretical chemists to quantitatively model the weak forces between organic molecules is being exploited to predict their crystal structures and estimate their physical properties. Evolving crystal structure prediction methods are increasingly being used to aid the design of organic functional materials and provide information about thermodynamically plausible polymorphs of speciality organic materials to aid, for example, pharmaceutical development. However, the increasingly sophisticated experimental studies for detecting the range of organic solid-state behaviours provide many challenges for improving quantitative theories that form the basis for the computer modelling. It is challenging to calculate the relative thermodynamic stability of different organic crystal structures, let alone understand the kinetic effects that determine which polymorphs can be observed and are practically important. However, collaborations between experiment and theory are reaching the stage of devising experiments to target the first crystallization of new polymorphs or create novel organic molecular materials.

## 1. Introduction

Most theoretical chemistry is based on Dirac's quote that ‘The fundamental laws necessary for the mathematical treatment of a large part of physics and the whole of chemistry are thus completely known, and the difficulty lies only in the fact that application of these laws leads to equations that are too complex to be solved’. Since Dirac's time, there have been huge advances in computer power, which has enabled a vast amount of research in making and testing approximate solutions to the laws of quantum mechanics, as recognized in the award of the Nobel Prize to Pople and Kohn in 1998. From the start of my PhD with Anthony Stone, I have been working from the more specific idea that you ought to be able to use quantum mechanics to determine models for the forces between molecules, and that, provided these were sufficiently accurate, you could then calculate all the physical properties of the molecules in the solid, liquid and gaseous states. Finding accurate enough analytical models for the forces between, as well as within, molecules has been one of the main limitations in the use of atomic-scale models, such as Molecular Dynamics (MD), for simulating the properties of condensed phases at finite temperatures. The power of such modelling, particularly in the study of biochemical processes, was recognized by the 2013 Nobel Prize to Karplus, Levitt and Warshel. This link between electronic structure and the dynamics of the nuclei, and the links, through multi-scale modelling, to the bulk properties of the molecules, should, in principle, allow us to predict the behaviour of specific molecules in all their phases. However, in trying to predict the crystallization behaviour of organic molecules, we keep on coming up against the questions that face all theoretical/computational chemists:

— what is a good enough approximate solution to the laws of quantum mechanics?
— what and how many atoms should be in the model for the system?
— what approximations can be made in the quantitative theory for the experimental data? Are the approximations being made in multi-scale modelling appropriate or are important molecule-specific details being omitted?

I have been fortunate in being able to collaborate with a broad range of experimental scientists on testing theories of how organic molecules, like pharmaceuticals, explosives and other functional molecules, crystallize. The aim is to develop a computational model capable of predicting how a given molecule would crystallize, and the physical properties of the crystals. If this can be done prior to the synthesis of the molecule, then the calculations could aid the design of new molecular materials. For molecules chosen for their intrinsic properties, such as pharmaceuticals, such calculations can reveal alternative ways of crystallizing and hence produce insights into the crystallization processes involved in pharmaceutical development. Pursuing this aim has shown that some of the approximations traditionally made in theories for the behaviour of molecules are not sufficiently realistic for useful computer predictions.

## 2. The theory of crystal structure prediction

The computational prediction of organic crystal structures (CSP) is based [1,2] on the theory that a molecule will crystallize in its most thermodynamically stable structure. This implies that predicting the crystal structure just requires generating all possible unit cells and evaluating their relative energies to find the most stable. This sounds simple, but when you come to implement it, you realize how many disciplines contribute to producing a computer code that can test this assumption.

The early search algorithms started with rigid molecules and assumed that there was only one molecule in the asymmetric unit, with the other molecules in the unit cell being generated by the space-group symmetry. Surveys of the Cambridge Structural Database (CSD) [3] when it contained less than a 100 000 crystal structures (*ca* 1990), showed that most organic molecules crystallized in a subset of the space groups, mainly with monoclinic and triclinic cells and some orthorhombic cells. There are far fewer possible space groups for a crystal of a chiral molecule containing only one enantiomer, as usually required for pharmaceuticals because of the possibility of different biological effects of a racemic mixture of the two mirror-image molecules. Further assumptions could be made to define a search that would generate only a few thousand plausible (i.e. reasonably close-packed, and low enough in energy in approximate models) crystal structures that needed more careful modelling of the relative thermodynamics. (CSP has always involved a compromise between the extent of the crystallographic space covered and the accuracy of evaluating the energies, resulting in a variety of hierarchical approaches.) Crystallographic technology has advanced enormously in recent years, with the ability to determine the atomic structure from smaller crystals and from crystalline powders, and now surveying the over 900 000 crystal structures in the CSD shows many structures with two or more independent molecules in the unit cell [4]. As the number of independent molecules increases, or the molecules have conformational flexibility, the number of variables in the search increases. Currently, searches are considering millions of possible structures, and generating tens of thousands of plausible structures and yet only a limited range of possible crystal structures have been covered. CSP provides a real challenge to optimization and search algorithms. In addition, the basic assumption that you can predict the crystal structure of a molecule by finding the most thermodynamically stable structure has been challenged by applying it to many molecules, particularly those where there has been an extensive experimental screen looking for possible polymorphs [5], i.e. alternative crystal structures containing just the same molecule.

**Figure 1.**
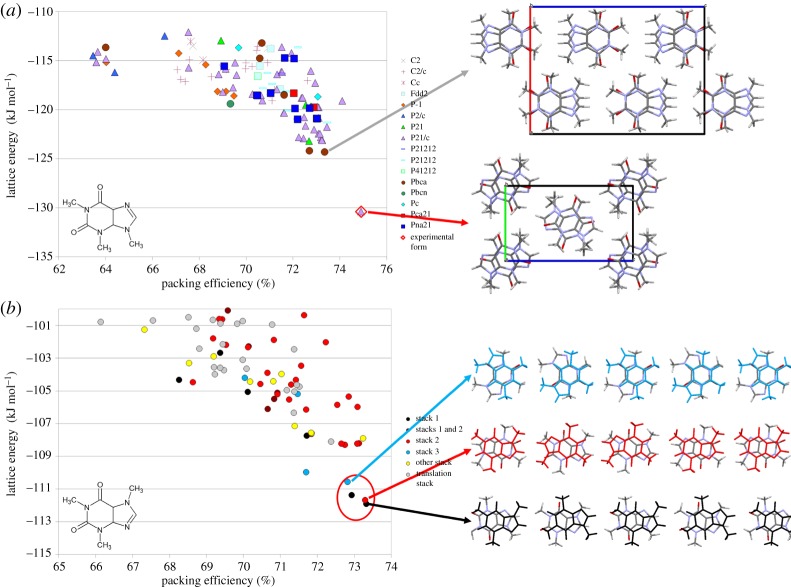
Example summaries of the output of a CSP study for the isomers (*a*) isocaffeine and (*b*) caffeine. Each symbol represents a mechanically stable crystal structure that is a minimum in lattice energy calculated by a *Ψ*_mol_ model, whose full three-dimensional crystal structure is classified by an appropriate property. The structures corresponding to experimentally observed forms are marked. A few selected crystal structures are illustrated in both cases. (*a*) An example of a large energy gap showing that only one crystal structure should be observed, and hence the default classification by space group of the structures is adequate [6]. The tilting of the molecules in the observed structure enables a slightly denser packing than any of the hypothetical computer-generated structures. (*b*) A case where a group of nearly equi-energetic structures typify the observed disordered polymorphs, in which the molecules lie parallel, and so the colour of the symbol indicates the type of stacking. Illustrated are three examples showing the stacking in the crystal of two chains of molecules, one in elemental colours, below the other in the colour associated with the type of stacking [6].

The assumption that the crystal structure would be the most thermodynamically stable form was anticipating that only one crystal structure would be observed, and that it would be sufficiently more stable than any others, that approximations in calculating the relative energies, such as the neglect of temperature, would not matter. Figure 1*a* shows one example with one of the larger energy gaps (about 6 kJ mol^−1^) between the observed structure and any other computer-generated possibilities that we have found in our studies of over 200 molecular systems. The energy gap is not much larger than the target chemical accuracy of quantum mechanical methods of 1 kcal mol^−1^ (4.2 kJ mol^−1^) and of the same order as the current state of the art for crystal energies; even for benzene, an absolute accuracy in the lattice energy of 1 kJ mol^−1^ has only recently been achieved [7]. There will be some cancellation of errors in relative crystal energies, but this will depend on the nature of the energetically competitive crystal structures. Unless there is only one, uniquely favourable mode of packing the molecules in all three dimensions, then there will be multiple structures that are competitive in energy. Crystal engineering can design hydrogen-bonding schemes and other motifs that are particularly favourable, such as chains or layers of molecules, but few organic molecules have unique strong directional interactions in three dimensions. This appears to explain the rarity of spontaneous resolution (where a mixture of both enantiomers of a chiral molecule separates on crystallizing to give enantiopure crystals), as a chiral column or layer of molecules can often pack with a centre of symmetry (producing a racemate) or without (producing an enantiopure (chiral) crystal) with very similar energies [8]. The observation that for most molecular systems, CSP generates more than one thermodynamically competitive crystal structure, reflects the experimental observation that most molecules are found to be polymorphic when crystallized under a wide range of conditions [5].

### (a) Polymorphism

The early CSP studies were academically motivated by fundamental scientific curiosity; we ought to be able to predict crystal structures if we understand crystallization and can model the forces involved. There was also a practical motivation, to avoid the expense of synthesizing energetic molecules, like octo-nitrocubane, that failed to crystallize in a dense structure, or nonlinear optically active molecules that crystallized in an inactive centrosymmetric structure. In those days, the first crystal of a molecule which could be grown to a size and quality that was suitable for X-ray diffraction was analysed for the structure of the molecule and was considered as the crystal structure. Then Abbott Laboratories lost control of the production of ritonavir, an anti-HIV treatment, because the most stable polymorph first appeared when the drug was being manufactured [9]. This reawakened the academic community to the phenomenon of polymorphism. Polymorphs contain the same molecule (as defined by the covalent bonding) in different crystal structures which have distinct physical properties. Solubility and dissolution rates are the most important physical properties for pharmaceuticals, but morphology, hydroscopicity, stability, light sensitivity, mechanical properties, etc., all affect the design of the pharmaceutical product, how it can be stored and the crystallization processes used in manufacture. Even when the active pharmaceutical ingredient (API) is not delivered in solid form, the dosage form still needs to be designed to ensure that it does not crystallize, as happened for the transdermal patches of rotigotine [10].

A huge effort has gone into devising automated and manual crystallization methods to attempt to establish all the possible polymorphs of a given API. The tendencies for metastable polymorphs to be found first (Ostwald's rule) [11–13] and for the crystallization of metastable forms to become much more difficult once a more stable form has been discovered (disappearing polymorphs) [14] add to the challenges of polymorph screening. The vast range of factors that have generated the first observation of new polymorphs, by changing the thermodynamic conditions or relative kinetics, with impurities often acting as a catalyst, makes exhaustive polymorph screening a practical impossibility. For pharmaceuticals, controlling the crystallization to produce phase pure samples in the licensed polymorphic form is essential for quality control. Hence the desire for a computer code that can predict all polymorphs, and ideally the crystallization conditions to find them [15–17].

**Figure 2.**
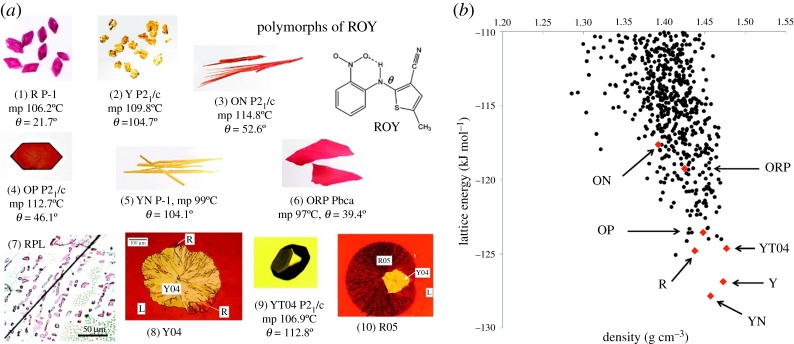
(*a*) The observed polymorphs of ROY (Reprinted from [18] Copyright 2010 American Chemical Society), with melting point, space group and conformational angle (*θ*) in the cases where a crystal structure has been determined, and (*b*) the output of a CSP study where the structures corresponding to the characterized forms are denoted in red, with black dots indicating the unobserved crystal structures that are also minima in the lattice energy. (Reprinted from [19]).

If the polymorph observed at a given temperature and pressure was always the most stable under those conditions, then the possible polymorphs would be on the calculated or experimentally determined phase diagram. However, the structurally characterized polymorphs of 5-methyl-2-[(2-nitrophenyl)amino]-3-thiophenecarbonitrile, also known as ROY because of the red, orange and yellow colours of its polymorphs [18] (figure 2), all exist at ambient temperature and pressure. Indeed, concomitant crystallization, where different polymorphs are observed in the same crystallization experiment, is sufficiently common that producing a powder X-ray diffraction pattern of a phase pure system is often a major barrier to solving a crystal structure. Polymorph screening may help design manufacturing processes to avoid concomitant crystallization and hence improve quality control. However, the metastable forms II and III of olanzapine have never been crystallized separately, despite extensive crystallization work when this anti-psychotic drug was under patent [20]. The barriers to a solid-state transformation depend on the molecule and the packing, with few systems transforming with changes in thermodynamic conditions as easily as benzene. Many polymorphs are enantiotropically related, i.e. the relative stability order changes within relevant temperature ranges, but this is often not experimentally apparent because of the slow speed of the first-order phase transition. The transition temperature is usually laboriously established by slurrying a sample of both polymorphs in a suitable solvent and determining which sample grows as the other dissolves [21].

The use of CSP as a complement to solid form screening for pharmaceuticals is in its infancy, but it has already been established that it would be useful to have the results available during the screening process [16]. The CSP low-energy structures can help with characterizing the structures of new polymorphs, by matching the predicted PXRD, electron diffraction or solid-state NMR of the CSP structures to the experimental results. Alternatively, the prediction of closely related structures, which often have very similar PXRD or vibrational spectra (e.g. eniluracil [22], thymine [23] and gallic acid monohydrate [24]), can raise the possibility of static or dynamic disorder in all or part of the molecule. For caffeine (figure 1*b*), the many closely related stacked structures point to the static disorder in the low-temperature phase and the dynamic disorder in the high-temperature phase [6]. For 3-(4-dibenzo[b,f][1,4]oxepin-11-yl-piperazin-1-yl)-2,2-dimethylpropanoic acid, a molecule that had been under development for treating sleeping disorders, there are two CSP-generated structures corresponding to form III which have indistinguishable power patterns as they differ only in the side-chain orientation. Since form III is formed by desolvating the hydrate, it is not surprising that it is a disordered structure with variable proportions of the two side-chain positions [25].

### (b) Testing the reliability of CSP methods

The progress of CSP and the development of the methodology have been charted by the blind tests organized by the Cambridge Crystallographic Data Centre [26], where groups are challenged to predict crystal structures (which crystallographers have contributed and kept secret) from their molecular diagrams. This has proved very valuable for the industrial scientists considering whether to invest in CSP, as well as being a community experiment that has spurred on innovations in methodology, and allowed an assessment of progress. Polymorphism inevitably complicates the analysis of the results of the blind tests, with some new polymorphs being found after the test was completed [24], though some of these polymorphs are outside the crystallographic criteria used in blind test targets (e.g. no disordered structures, *Z′* = 1). The molecular structure is no guide to the crystallization behaviour: for example, most methods would correctly identify the crystal structure of isocaffeine (figure 1*a*), but none could predict the crystal structures of its isomer caffeine (figure 1*b*), whose known forms are crystallographically disordered [6]. The recent Faraday Discussion on CSP shows the huge progress in the field [17], and that it is arguably becoming an applied technology [27]. However, even the basic assumption behind the computer codes, that the molecule will adopt the most thermodynamically favourable crystal structure, is open to question [28], and some molecules do not appear to crystallize at all. The questions about which structures are metastable kinetic products, and which are the thermodynamically most stable at a specified temperature and pressure, requires considerable advances in our theoretical methods, let alone the challenge of calculating all the properties of the polymorphs that would be needed in the design and manufacture of an industrial product.

## 3. Challenges to theory for calculation of accurate enough relative energies of crystals

Crystallizing the most stable polymorph under the conditions of temperature and pressure that the material will be used is the safest strategy to avoid the risk of conversion. However, predicting the most stable polymorph requires modelling the free energy, rather than just the static lattice energy. This is a major computational challenge, as demonstrated by the recent calculation of the polymorph phase diagram of methanol [29]. Realistically including the differential effects of temperature on polymorphs is challenging as most organic crystals at ambient temperature are nearer to their melting points than 0 K, or decompose before melting. Thermal expansion is significant, often very anisotropic, and even zero-point motions within crystals can affect the volume [30] by a few %. Hence there is a limit to the accuracy with which the crystal structure can be predicted by lattice energy methods that assume a static lattice. The relative free energies are much more challenging to calculate than the structure; a few crystal structures can be predicted by close packing a hard-sphere model of the van der Waals surface to fit bumps into hollows [31]. The phonon density of states is very dependent on the crystal structure, and this can give rise to differences in heat capacities between polymorphs or between the racemic and enantiopure crystals of the same molecule [32]. Hence, while relative lattice energies of the racemic and enantiopure crystal (which could be estimated as the global minimum in the CSP in the relevant space group) can be sufficiently large to show that chiral separation by crystallization is impossible, it is not yet possible to predict the relative solubilities as a function of temperature or solvent accurately enough for aiding the design of such processes [33]. Modelling free energies of organic crystals is currently a major and challenging area for method development, either by developing lattice dynamics methods that reflect the coupling between lattice and molecular modes and include thermal expansion [34] or by deriving free energy differences from molecular dynamics simulations. Comparisons between different approaches are rare [35]. Fortunately, in the majority of known polymorphic systems, the relative stability order can be calculated correctly by ignoring the differential effects of the molecular motions in the crystals [36].

Most CSP methods approximate the free energy by the lattice energy, the energy required to take a static model of the crystal (0 K, ignoring zero-point motion) and separate it into isolated static (gas-phase) molecules in their lowest energy conformation. This has become a major application of periodic Density Functional Theory (DFT), seeking increasingly accurate approximations to solve the electronic structure of the crystal [37]. Organic crystals represent a significantly greater challenge than inorganic (ionic) crystals, as there is a much larger range of interatomic forces involved, from the covalent bonding within the molecule to the much weaker intermolecular forces. These forces are very anisotropic, with different polymorphs representing different compromises between the different types of inter- and intramolecular forces. In certain molecular crystal structures, the interactions in one crystallographic direction could be dominated by intermolecular dispersion, the universal, non-classical van der Waals force that is responsible for the crystallization of argon. These dispersion forces arise from the correlation of electron motion between the molecules, and so are not calculated at all by many approximate quantum mechanical theories. Intermolecular forces (and intramolecular forces in larger flexible molecules [38]) are affected by basis set superposition error, and so need large basis sets to converge the calculation. The DFT functionals that can be afforded for periodic electronic structure calculations on even just a few dozen different CSP-generated crystal structures are limited in accuracy; there is an increasing range of dispersion corrections being developed (periodic DFT + D methods) and applied to molecular crystals [39]. Currently, the relative stability ranking of the lowest energy CSP-generated crystal structures can depend on the dispersion correction used. Hence molecular crystals of increasing size represent a challenge to developing electronic structure theory of solids, even at the level of the lattice energy, let alone also modelling the free energies by lattice dynamics. We use *Ψ*_crys_ to denote the general approach where electronic structure calculations are used on each crystal structure, covering periodic molecular orbital wavefunction methods and density functional approaches, and fragment-based methods which do calculations on all dimers and trimers, etc., in each crystal structure. Improving the relative accuracy, speed and dependence on the number of atoms in the largest unit cells is a major area of research in the competing methods [40]. As computers get faster, more accurate methods can be applied to the molecular crystals of increasingly large molecules. However, CSP approaches are inevitably hierarchical, with increasingly accurate methods being applied as millions of plausible crystal structures are reduced to those within the energy range of being plausible polymorphs. This energy range is not an absolute, though commonly taken as 5–10 kJ mol^−1^. For example, polymorphs of pharmaceuticals that are produced by desolvating solvated crystal structures may be trapped as highly metastable forms because of the high barriers to rearrangement in the solid state.

The alternative approach, which we have been using, started from modelling rigid organic molecules and calculating the charge density of the molecule (*Ψ*_mol_), to derive the molecular structure and a distributed multipole representation of the molecular charge density [41]. Dipoles and quadrupoles, as well as charges on each atom, can be used in an anisotropic atom–atom model for the electrostatic interactions which thereby includes the anisotropic effects of lone pair and π electron density on the intermolecular interactions. This provides a more directional and realistic model of hydrogen bonding, π … π stacking, halogen interactions, etc. This proved to be an essential improvement for modelling the relative lattice energies of organic crystals [42], which are usually close-packed compromises between the different types of intermolecular interactions. The development of anisotropic atom–atom intermolecular potentials from the wavefunction of the molecule using the theory of intermolecular forces [41] has been the theme of my research since my PhD. The program ORIENT [43], which uses such potentials for modelling clusters of rigid molecules or molecular clusters on molecular surfaces [44,45], has been developed by Anthony Stone and collaborators since the 1980s when it first demonstrated how important distributed multipoles were for predicting the structures of van der Waals dimers [46]. The equations for the associated non-central forces and torques are complex [47]. My group has been developing the program DMACRYS for modelling crystals of rigid organic molecules [48] since the early 1990s. Methods of deriving the other contributions to the intermolecular forces, the long-range induction and dispersion energies from distributed polarizabilities, and all the short-range terms using symmetry-adapted perturbation theory, have been developing rapidly [49]. This is leading to the development of non-empirical model intermolecular potentials in an anisotropic atom–atom form, such as the CamCASP approach [50]. These model potentials allow quite accurate energies and properties to be calculated very rapidly for use in CSP, and completely non-empirical potentials have been used recently for C_6_Br_2_ClFH_2_ [51] and pyridine [52]. This approach is capable of generating the best atom–atom intermolecular potentials for a specific molecule [52], being limited by the assumed functional form and assumptions made in the terms included and monomer and dimer calculations used in the parametrization.

These non-empirical potentials are a welcome improvement on the empirically parametrized isotropic exp-6 potentials that have mainly been used in CSP [48], which are nevertheless comparable in accuracy with some popular *Ψ*_crys_ DFT + D methods [53]. The empirical parametrization absorbs many errors in the functional form of the force-field, such as thermal expansion effects or missing polarization and many body terms. Hence empirically parametrized model potentials can only be expected to behave well for related molecules and their properties used in fitting the potentials, such as crystal structures and heats of sublimation. Thus, only a non-empirical potential could be used to identify a new high-pressure polymorph of pyridine [52] as it had a more realistic extrapolation high up the repulsive wall than the empirical potential. However, it (correctly) gave a lattice energy minimum structure that was slightly denser than even the 5 K crystal structure, because of the neglect of zero-point motion on the crystal structure and three body dispersion terms. A truly accurate (electronic) potential energy surface would require the addition of the nuclear dynamics at a quantum level to give agreement with experiment for cases, like pyridine, where the polymorph behaviour changes with deuteration [54].

The *Ψ*_mol_ method is very effective for rigid molecules, but most organic molecules are not rigid, with at least some torsion angles that are likely to change to improve the intermolecular contribution to the lattice energy, even when this gives a higher energy conformation. These energy changes are often small but significant in improving the energy. However, in cases, for example, where an intramolecular hydrogen bond in the isolated molecule becomes intermolecular in some polymorphs, the conformational energy penalty can be quite large. Thus, CSP for flexible molecules requires an accurate balancing of the model for the intermolecular and intramolecular forces so that polymorphs with very different hydrogen bonding or π…π stacking are modelled well, which is testing of the basic quality of the either the *Ψ*_crys_ or *Ψ*_mol_ methods. *Ψ*_crys_ methods have the advantage that, as they do not separate the atoms into molecules, all conformational degrees of freedom can be simultaneously optimized, though the differences between covalent and dispersion-dominated interactions makes the surface complex for optimization. The *Ψ*_mol_ method requires identifying the conformational degrees of freedom that drastically change the shape and interactions of the molecule, as these have to be considered in the generation of crystal structures, and identifying others that can vary slightly but have a marked effect in the refinement of crystal structures and energies. Algorithms and databases of the *Ψ*_mol_ calculated properties make this approximation quite efficient for molecules with relatively few flexible conformational degrees of freedom [55].

The approaches to CSP that have reasonable success rates in the blind tests of CSP [26] rely on quantum mechanical, electronic structure calculations covering either the range of possible molecular conformations within crystal structures (*Ψ*_mol_), or the range of low-energy crystal structures (*Ψ*_crys_). It is notable that traditional transferable isotropic atom–atom force-fields, designed for Molecular Dynamics simulations, are rarely sufficiently realistic to be able to get the observed crystal structures lower or reasonably comparable in energy with those generated in a CSP study. This can be attributed to the inadequacy of the assumptions that the same charges and non-bonded terms can be used within and between molecules, that the atoms interact as if they were spherical within molecules, and that parameters can be transferred between chemically similar molecules. The limitations of traditional transferrable force-fields are well known, with the ‘next generation’ of biological force-fields [56] using multipolar electrostatics and considering polarization and transferability assumptions. Molecule-specific force-fields can be accurate enough to reduce the number of *Ψ*_crys_ calculations needed, as in the approach used by Avante-Garde Materials Simulation [57]. Analysis of the conformational profiles of some fenamates suggests that force-fields for pharmaceuticals could be significantly improved by fitting a separate atom–atom intramolecular force-field [58]. Hence, CSP is a useful severe test for force-field development. It is also a field which needs accurate enough atomistic force-fields that temperature effects can be modelled realistically enough to remove structures that are not free energy minima at practically relevant temperatures [59,60].

## 4. Predicting properties of organic crystals

The CSP process generates the crystal energy landscape, the structures and estimated energies of the structures that are thermodynamically plausible as polymorphs. Many other properties can be calculated from the structures. The most widespread use of CSP structures is to help structurally characterize polymorphs in cases where the available experimental data are insufficient, most commonly when it is not possible to grow a single crystal suitable for diffraction measurements. A CSP-generated structure can provide an initial model for solving a structure from powder diffraction data [61], though the match of simulated and experimental data is often complicated by the temperature dependence of the peak positions, phase purity, preferred orientation effects and errors in predicted cell dimensions. CSP can show that multiple structures can match the powder X-ray pattern. This raises the question as to whether the sample is one disordered phase or a mixture of phases [25]. A rarer example was in characterizing a new polymorph of theophylline by electron diffraction, when it was spotted by its distinctive morphology, though present below the threshold for detection by powder X-ray diffraction [62]. The calculation of the solid-state NMR spectrum from the crystal structure is producing a new field of NMR determination of crystal structures [63].

The second group of properties are those affecting processing, such as solubility, morphology and mechanical properties. Here there is an evolving hierarchy of methods under development, involving different approximations. The relative solubilities of polymorphs are an important property for pharmaceutical development, including separating the different enantiomers of chiral molecules by crystallization using the solubility difference between enantiopure and racemic crystals [33]. It was once thought that relative lattice energy differences (using the CSP lowest energy when the crystal structures are not known) might be sufficient for the design of diastereomeric resolving agents or prediction of spontaneous resolution. However, the exponential dependence of solubility differences on the free energy differences makes this a very hard property to predict when such separation is possible, particularly if there is a marked temperature dependence to the differential heat capacity [32]. Morphology is another important property for processing, and the attachment energy model allows prediction of morphologies (and relative crystal growth rates) from the crystal structures, in a theory that is only strictly valid for vapour grown crystals below the roughening temperature. Models that take into account the effect of solvent and use a more realistic mechanism are now emerging [64]. The elastic properties of a perfect infinite crystal can be calculated within the harmonic approximation [65], but the effects of dislocations, grain boundaries and other inevitable crystal imperfections on the elastic properties are more marked than on the other second derivative properties associated with the phonon modes.

The final group of properties are those of functional organic materials. The ability to calculate the property of interest, be it gas absorption, conductivity, nonlinear optical coefficient or explosive properties, from the crystal structure, is key to producing structure–property crystal landscapes, to see whether the molecule might crystallize in a structure with worthwhile properties. It also shows the extent to which the property can vary among the possible polymorphs, indicating the importance of polymorph control in manufacture.

## 5. Why does CSP often over-predict polymorphism?

Occasionally a CSP study confirms that all thermodynamically plausible structures are known, but it is more common that there are many more lattice energy minima than known or likely polymorphs (e.g. figure 2*b* [19]), raising the question, why don't we find more polymorphs? [66] Many are artefacts of the neglect of temperature as distinct lattice energy minima may correspond to the same free energy minimum. However, predicting which of the other structures may be crystallized and not readily transform to a more stable polymorph requires an understanding of competitive nucleation, growth and transformation rates that is only slowly beginning to emerge.

It is highly likely that closely related CSP-generated crystal structures would be indistinguishable during crystallization, particularly if the crystallization does not go via classical nucleation theory where the nucleus has the final structure, but via a two-step process involving a densification of the solute prior to crystallization. Indeed, the hydration of olanzapine has been observed to proceed by the growth of dense droplets at the ledges on the surface of olanzapine crystals, where there are many favourable binding sites that are not the crystallographic site. Which polymorph of the hydrate is formed depends on whether this droplet remains in contact with the surface, or whether it is detached by stirring [44]. The relative thermodynamic driving force can change during a cooling crystallization, and the metastable zone-widths of the competing forms may also differ in some solvents, making the polymorphic outcome very sensitive to the details of the crystallization conditions.

Sometimes, closely related structures are seen but as components of disorder or stacking faults [67]. Aspirin form II, an early case where the CSP-generated structure was in the literature before it was observed, differs from form I in the stacking of the same hydrogen-bonded sheets. Domains of each polymorph can be seen in the same single crystal [68]. The third polymorph of aspirin has recently been found and characterized with the aid of CSP [69]. Stacking disorder that seemed likely from the CSP generating structures with different stackings of the same sheets, was sufficient within all three racemic polymorphs of tazofelone to give a variation in the melting points of single crystals and show as streaking in the diffraction data [70]. The difference between the nucleation and growth kinetics resulting in high-quality single crystals or disordered powders with stacking errors depends on the ability to correct growth errors before they are incorporated into the lattice. The longevity of a metastable polymorph will be very dependent on the structural defects and crystal size: for example, the polymorphs of 1,2,4,5-tetrachlorobenzene are very similar, but the reversible transformation with temperature shows considerable hysteresis in powder diffraction experiments and was not seen in a single crystal without any twin domain boundaries [71].

The relative stability of polymorphs can depend on the size of the crystal [72] and solvent at nanoscale sizes where the proportion of surface to bulk molecules is not negligible. This can help explain why confinement in nano-pores or droplets can affect the polymorph formed [73]. However, the observation of metastable polymorphs in tiny droplets can just reflect the inhibition of the transformation or lack of replenishment of the supersaturation [74]. We are only just beginning to see why so many factors such as solvent, agitation, vessel design, cooling rates, removal from liquor, confinement, heterosurfaces and impurities (or other molecules in failed cocrystallization experiments) can sometimes lead to different polymorphs.

So, when CSP predicts more possible polymorphs than are known, are they an artefact of your computer model (and we know why many could be), or are they polymorphs that have not yet been found? What experiment might be proposed to find a specific polymorph? The use of crystallization under pressure [75] to target polymorphs that CSP suggests are denser and of increasing relative stability with pressure has been successful [76]. More specifically, if the targeted structure is similar to that adopted by another (closely related) molecule, then heteromolecular seeding can produce the predicted form [77]. Since solid solutions may also be formed if molecules can adopt very similar structures, sublimation onto a templating crystal can be a more effective method of generating the first sample of a new polymorph [78]. Having a computer-generated structure that might be a potential polymorph is a sign that the experimental polymorph screening may not be complete, but calculating the properties of the hypothetical crystal may suggest experiments to find it.

Understanding how different surfaces, solvents, etc., can affect which polymorph crystallizes is an area of active experimental and simulation research, varying from tackling the challenges of experimental observation, e.g. by cryo-transmission electron microscopy [79], to simulating pre-critical fluctuations on different idealized surfaces [80]. Combined experimental and computational studies of nucleation on surfaces will further emphasize how the shape and intermolecular interactions of the specific molecule can vary the nucleation and crystal growth behaviour [81].

There is a lot of work to do before we really understand crystallization well enough to find all polymorphs and reproducibly control their formation. Thermodynamics is always involved: if you are targeting a metastable polymorph, there needs to be a set of conditions in which it will nucleate and grow faster than the stable form, and be detected and characterized before it has transformed. Even if a form is thermodynamically the most stable, it may not be practically important if it has a relatively slow growth rate, and so samples will not seed an efficient crystallization. In one of the rare cases (to date) where CSP correctly lead to the discovery of the most thermodynamically stable form, it was difficult to both nucleate and grow in competition with the previously known form [82].

Organic molecules with awkward shapes and competing intermolecular interactions are very different from the isotropic spheres assumed in the development of classical nucleation theory and crystal growth. Organic crystals rarely, if ever, undergo second-order solid-state phase transitions, even when the structures are closely related. Hence, molecular dynamics simulations, with modifications to sample the rare events that are involved in crystallization and transformations, are going to be necessary to get the insights into how the kinetics of these processes dictate which of the CSP-generated thermodynamically plausible polymorphs are practically important.

## 6. The way forward

Control and prediction of the organic solid state remains a fundamental and practical challenge, but one where multi-scale modelling, from using electronic structure theory through to predicting crystal structures and their bulk material properties, is showing promise. The goal of producing computer-aided guidance for the design of new materials, to avoid the synthesis of molecules that would not form crystals with the desired physical properties, is progressing in various fields of organic functional materials. The successful design of a molecule that did crystallize a highly porous crystal for gas storage [83] exemplifies the progress that can be made through close collaboration between theory and experiment. Theoretical predictions of crystal structures and properties are rapidly progressing to being capable of aiding experimental work in the design of organic electronic, optical and energetic materials. The ability to predict the thermodynamically plausible polymorphs of a given molecule is becoming a useful aid to experimental screening for characterizing new polymorphs. Sometimes the calculations can suggest additional experiments to find new forms or help rationalize the crystallization behaviour of a molecule. Thus, the exploitation of advances in computer power to allow approximate solutions to the laws of quantum mechanics to predict or rationalize organic crystal structures and their properties has reached a state of accuracy that allows very productive collaborations between theory and experiment. However, there is still a long way to go before such theoretically based modelling methods can tackle the range of size of molecules and properties with the accuracy required.

The basic CSP technique to see in which ways a molecule can pack with itself to form a crystal structure still raises many fundamental questions. Will a molecule crystallize at all, let alone in the most thermodynamically stable form (as currently assumed)? There is a lot more we need to understand about the nucleation and growth of organic crystals before we can devise experiments to help find all relevant polymorphs and improve the design and quality control in manufacturing organic speciality materials.

## Data accessibility

Not applicable. The CSP-generated crystal structures in the authors published work since 2004 are available in .res format on request.

## Author profile

Sarah (Sally) Price FRS FRSC is Professor of Physical Chemistry at University College London. After completing a BA in Natural Sciences and a PhD in theoretical chemistry under the supervision of Anthony Stone at the University of Cambridge, she went to the University of Chicago before returning to Cambridge to continue work on modelling intermolecular forces. She was awarded a Royal Society University Research Fellowship in 1985, which was transferred to University College London in 1989 and included maternity leave for her two children. At UCL she started to develop the theory and computer codes to model the organic solid state using anisotropic atom–atom intermolecular model potentials. Sally has collaborated widely with experimental solid state chemists, pharmaceutical scientists, chemical engineers, theoretical physicists and computational scientists, including leading the Basic Technology Project 'Control and Prediction of the Organic Solid State'. She was awarded the RSC Interdisciplinary Prize in 2015 and elected to the Fellowship of the Royal Society in 2017 in recognition of the value of this collaborative computational work in revealing the complexities of organic crystallization.
